# *De novo* transcriptome sequencing of axolotl blastema for identification of differentially expressed genes during limb regeneration

**DOI:** 10.1186/1471-2164-14-434

**Published:** 2013-07-01

**Authors:** Cheng-Han Wu, Mong-Hsun Tsai, Chia-Chuan Ho, Chien-Yu Chen, Hsuan-Shu Lee

**Affiliations:** 1 Institute of Biotechnology, National Taiwan University, Taipei, 106, Taiwan; 2 Graduate Institute of Biomedical Electronics and Bioinformatics, National Taiwan University, Taipei, 106, Taiwan; 3 Department of Bio-Industrial Mechatronics Engineering, National Taiwan University, Taipei, 106, Taiwan; 4 Department of Internal Medicine, National Taiwan University Hospital and National Taiwan University College of Medicine, Taipei, 100, Taiwan; 5 Agricultural Biotechnology Research Center, Academia Sinica, Taipei, 115, Taiwan; 6 Research Center for Developmental Biology and Regenerative Medicine, National Taiwan University, Taipei, 100, Taiwan

**Keywords:** *Ambystoma mexicanum*, Axolotl, Transcriptome, Blastema, *De novo* transcriptome sequencing

## Abstract

**Background:**

Salamanders are unique among vertebrates in their ability to completely regenerate amputated limbs through the mediation of blastema cells located at the stump ends. This regeneration is nerve-dependent because blastema formation and regeneration does not occur after limb denervation. To obtain the genomic information of blastema tissues, *de novo* transcriptomes from both blastema tissues and denervated stump ends of *Ambystoma mexicanum* (axolotls) 14 days post-amputation were sequenced and compared using Solexa DNA sequencing.

**Results:**

The sequencing done for this study produced 40,688,892 reads that were assembled into 307,345 transcribed sequences. The N50 of transcribed sequence length was 562 bases. A similarity search with known proteins identified 39,200 different genes to be expressed during limb regeneration with a cut-off E-value exceeding 10^-5^. We annotated assembled sequences by using gene descriptions, gene ontology, and clusters of orthologous group terms. Targeted searches using these annotations showed that the majority of the genes were in the categories of essential metabolic pathways, transcription factors and conserved signaling pathways, and novel candidate genes for regenerative processes. We discovered and confirmed numerous sequences of the candidate genes by using quantitative polymerase chain reaction and *in situ* hybridization.

**Conclusion:**

The results of this study demonstrate that *de novo* transcriptome sequencing allows gene expression analysis in a species lacking genome information and provides the most comprehensive mRNA sequence resources for axolotls. The characterization of the axolotl transcriptome can help elucidate the molecular mechanisms underlying blastema formation during limb regeneration.

## Background

*Ambystoma mexicanum* (axolotl), one of over 500 species of salamander, can completely reconstitute lost limbs after amputation. The amputation of limbs results in the formation of blastemas in the stump ends. These blastemas contain undifferentiated cells capable of growing and developing into new limbs exactly as they were before amputation [1]. In the early phase of regeneration, growing wound epithelium and epidermis cover the ends of the truncated nerves and the surface of the amputation site within several hours [2-4]. After the nerves and wound epidermis contact each other, the epidermis overlying the axon ends thickens, forming an apical epithelial cap [5]. Fibroblasts from the surrounding tissue simultaneously migrate to the amputation site under the apical epithelial cap. These fibroblasts proliferate to form a mass of undifferentiated cells that subsequently develops into the new limb. In the absence of functional nerves, an apical epithelial cap and blastema cannot be formed on the amputation stump [6]. Instead, denervated limbs undergo a wound-healing response post-amputation, and do not regenerate [7,8].

In past several years, next-generation sequencing (NGS) technology has become a cutting-edge approach for high-throughput sequence determination. This technology has dramatically improved the efficiency and speed of gene discovery in many studies [9,10], and has significantly accelerated and improved the sensitivity of gene expression profiling. For example, studies in the field of human [11] and *Arabidopsis*[12] transcriptomics have made remarkable progress. Studies using transcriptome sequencing for organisms with complete genome sequencing have confirmed that the short-read products of NGS can be effectively assembled and used for gene discovery and comparison of gene expression profiles.

National Center for Biotechnology Information (NCBI) [13-15] and Sal-Site [16] have already made available many cDNA sequene databases for axolotl. However, there still exist many unannotated protein-coding genes or non-coding RNA sequences that have not been sampled previously and are not present in existing cDNA libraries. Recent reports have also suggested that more *Ambystoma* to human non-redundant (nr) orthologous sequences remain to be discovered [15]. Moreover, sequence coverage of transcripts is highly variable between different cDNA libraries. With more available cDNA sequences, the overall sequence coverage of axolotl transcripts will be improved. Although previous studies have highlighted the usefulness of cDNA sequencing for the discovery of candidate genes in the absence of a genome sequence database, a comprehensive description of the full spectrum of genes expressed in axolotl blastemas is still lacking. To our knowledge, the genome sequencing of any salamander species has not been completed.

Several studies have used highly parallel 454 pyrosequencing to identify axolotl sequences which are used to generate a large-scale feature axolotl microarray [14,15,17,18]. However, 454 pyrosequencing has relatively lower overall transcriptome coverage when compared to Illumina/Solexa platforms [19-21]. Several recent studies have employed the Illumina/Solexa platform to offer a far greater coverage than 454 pyrosequencing [19-21]. However, in the early stages of this platform, the majority of Illumina sequence reads could not be matched to known genes because of their short length. In general, 454 pyrosequencing had longer sequence reads whereas Illumina sequencing had shorter, but more numerous paired ends read [19-21]. Currently, the latest developments in 454 and Illumina technologies offer higher resolution and are relatively consistent with each other. With improved quality and longer reads, the higher coverage from Illumina technologies allows for the identification of low-abundance genes not detected in earlier studies of limb regeneration based on 454 pyrosequencing. Therefore, Illumina platforms are well suited for gene discovery and promising insights into axolotl limb regeneration.

The *de novo* transcriptome sequencing of axolotl blastema in this study produced over 4 billion bases of high-quality cDNA sequences, which were assembled and annotated without a reference genome. 116,787 distinct sequences, including hundreds of developmental genes and wound-healing genes were identified. The gene expression profiles of a regenerating blastema and a non-regenerating denervated limb stump, 14 days post-amputation, were compared using differential gene expression analysis. A list of genes significantly overexpressed in normal regenerating blastema was obtained from the results of the analysis. A subset of genes from this list was verified using quantitative polymerase chain reaction (qPCR) and *in situ* hybridization.

## Results and discussion

### Illumina NGS and read assembly

To obtain the gene expression profiles of an axolotl blastema and a non-regenerating denervated limb stump, mRNA samples of both types of tissue, 14 days post-amputation, were prepared and sequenced using an Illumina sequencing platform. We performed a single sequencing run for each of the 2 tissues. After cleaning and quality assurance, we obtained 40 million 100-bp reads from both samples (Table 1). These raw reads were randomly clipped into 21-mers for sequence assembly using SOAP de-novo [22] to yield 2,920,951 contigs (Table 1) with a mean contig size of 117 bp. The size distribution of these contigs is shown in Additional file 1. In a previous report [14], Monaghan et al. generated over 1.7 million reads and approximately 400,000 unique sequences with a mean contig size of 215 bp for axolotl from a broader range of regeneration stages using 454 pyrosequencing. Using paired-end joining and gap-filling, we further assembled these contigs into 307,345 transcribed sequences with a mean size of 373 bp including 20,504 transcribed sequences larger than 1000 bp (Table 1). After clustering using TIGR Gene Indices clustering tools (TGICL) [23], the 307,345 transcribed sequences generated 116,787 unigenes with a mean size of 529 bp (Table 1). In this report, the term “unigene” indicates a transcribed sequence that matches no other transcribed sequence and has distinct sequences.

**Table 1:** **Summary for *Ambystoma mexicanum *transcriptome**

Total number of reads	40,688,892
Total base pairs (bp)	4,862,000,340
Average read length	100 bp
Total number of contigs	2,920,951
Mean length of contigs	117 bp
Total number of transcribed sequences	307,345
Mean length of transcribed sequences	373 bp
Transcribed sequence N50	562 bp
Total unigenes	116,787
Mean length of unigenes	529
Unigenes with E-value < 10^-5^	39,200

The data sets are available at the NCBI Short Read Archive (SRA) with the accession number: SRA064951.

### Annotation of predicted proteins

**Figure 1:**
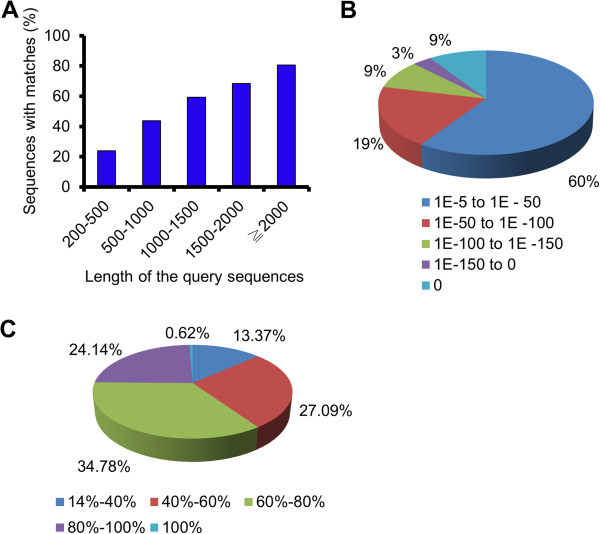
**Characteristics of homology search of Illumina sequences against the nr database. (A)** Effect of query sequence length on the percentage of sequences for which significant matches were found. The proportion of sequences with matches (with a cut-off E-value of 1.0E-5) in the NCBI nr databases is greater among the longer assembled sequences. **(B)** E-value distribution of BLAST hits against the nr database for each unique sequence with a cut-off E-value of 1.0E-5. **(C)** Similarity distribution of the top BLAST hits for each sequence.

For annotation, BLASTx was used to compare unigenes against the NCBI nr database by using a cut-off E-value of 10^-5^. Using this approach, 39,200 genes (33.6% of unigenes) were found to be above the E-value cut-off (Additional file 2). Because of the relatively short length of unigene sequences (mean size of 529 bp) and lack of genome reference in axolotl, most of the 77,587 (66.4%) assembled sequences could not be matched to any known genes. Figure 1A shows that the proportion of assembled sequences with matches in the nr database is higher among the longer sequences. Specifically, sequences longer than 2000 bp had a match efficiency of 80.6%, whereas the match efficiency decreased to approximately 43.8% for sequences ranging from 500 to 1000 bp in length, and to 23.9% for sequences between 200 and 500 bp in length (Figure 1A). The E-value distribution of the top matches in the nr database showed that 40% of the mapped sequences have strong homology (smaller than 1.0E-50), whereas 60% of the homologous sequences ranged from 1.0E-5 to 1.0E-50 (Figure 1B). The sequence similarity distribution has a comparable pattern, with 24.8% of the sequences having a similarity higher than 80%, and 75.2% of the sequences having a similarity ranging from 14% to 80% (Figure 1C).

### Gene ontology and classification of clusters of orthologous groups

**Figure 2:**
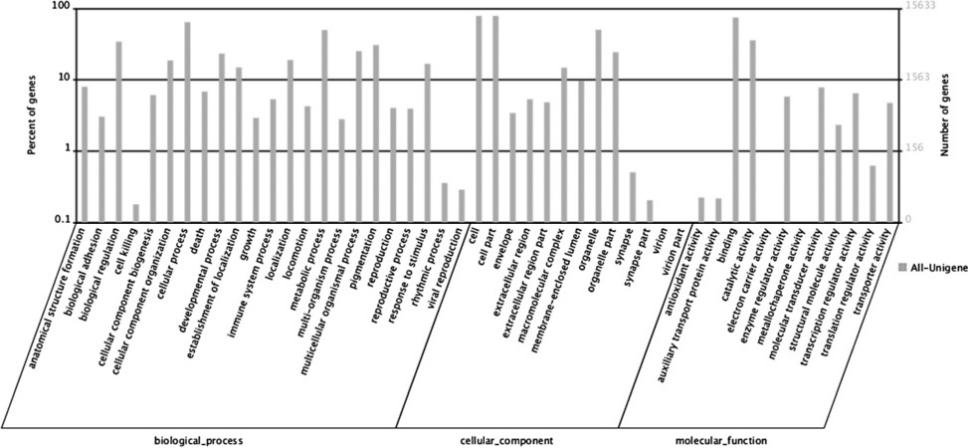
**Histogram presentation of Gene Ontology classification.** Results are summarized in three main categories: biological process, cellular component, and molecular function. The right y-axis indicates the number of genes in a category. The left y-axis indicates the percentage of a specific category of genes in the main category.

**Figure 3:**
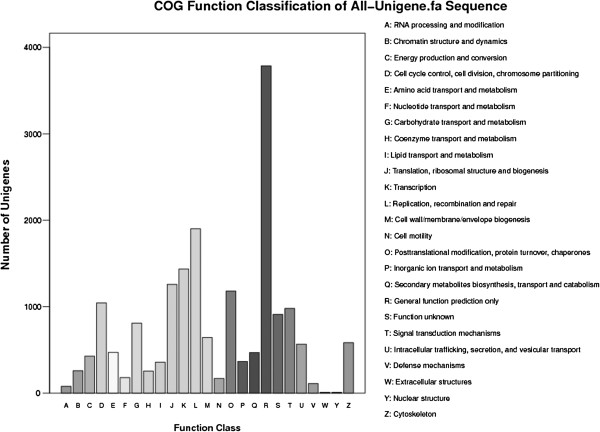
**Histogram presentation of clusters of orthologous groups (COG) classification.** Out of 39,228 nr hits, 9744 sequences have a COG classification among the 25 categories.

Gene ontology (GO) assignments were used to classify the functions of the predicted axolotl genes. Based on sequence homology, 15,633 sequences can be categorized into 48 functional groups (Figure 2). In each of the 3 main categories of GO classification (i.e., biological process, cellular components, and molecular function), “cellular process”, “cell part”, and “binding” terms are dominant, respectively. However, we found a few genes in the categories: “virion”, “virion part”, “metallochaperone activity”, and “electron carrier activity”. We also noticed a high percentage of genes in the “developmental process” and “response to stimulus” categories, but only a few genes for “cell killing” and “antioxidant activity”. To further evaluate the completeness of this transcriptome library and the effectiveness of the proposed annotation process, we searched the annotated sequences for the genes involved in clusters of orthologous groups (COG) classifications. Of the 39,200 matches to the nr database, 9744 sequences have a COG classification (Figure 3). Among the 25 COG categories, the cluster for “general function prediction” represents the largest group (3785, 20.7%), followed by “replication, recombination, and repair” (1901, 10.4%) and “transcription” (1436, 7.9%). The following categories represent the smallest groups: “extracellular structures” (9, 0.049%), “nuclear structure” (7, 0.049%), and “RNA processing and modification” (79, 0.43%) (Figure 3). To identify the biological pathways active in the axolotl, we mapped unigenes to canonical reference pathways using the Kyoto Encyclopedia of Genes and Genomes (KEGG) [24]. In total, 24,095 sequences were assigned to 217 KEGG pathways. The pathways with the most representation by the unique sequences were starch and sucrose metabolism (2801 members, 11.62%), pathways in cancer (1126 members, 4.67%), and regulation of actin cytoskeleton (1029 members, 4.27%). These annotations provide a valuable resource for investigating specific processes, functions, and pathways in axolotl limb regeneration research.

### Detection of growth factor and transcription factor sequences related to limb regeneration in axolotls

Fibroblast growth factors (FGFs) participate in salamander limb regeneration [25-29]. For example, *fgf*-*8* is expressed in the basal layer of the apical epithelial cap [30]. Developmental genes are also re-expressed in blastema mesenchymal cells [31-36]. For example, *homeobox A13*, an autopod marker in many vertebrates, appears in the distal region of the blastema [32,37,38]. Therefore, sequences related to growth factors and transcription factors involved in regeneration were analyzed and compared to sequences from NCBI nucleotides and the EST database. As Table 2 shows, this process identified a number of sequences that are homologous to growth factors related to limb regeneration, such as *transforming growth factor* (*tgf*)-*βs* and *fgfs*, as well as transcription factors involved in limb regeneration, such as *Homeobox* (*hox*) genes. In total, this study identified 6 *tgf*-*β* sequences. After removing redundant sequences, 3 different *tgf*-*β* isoforms, 17 different *hox* genes, and 7 different *fgfs* were identified (Table 3). Four *tgf*-*βs* (*tgf*-*β1*, *2*, *3*, and *5*) have been identified previously in axolotl [39]. This study identifies 3 (*tgf*-*β1*, *2*, *3*) and represents a nearly complete collection of such genes in axolotl. These findings confirm the high quality and high yield of the sequencing data in this study, and may facilitate axolotl limb regeneration research.

**Table 2:** **Comparison of the sequence numbers of transforming growth factor**-***β*s**, **fibroblast growth factors**, **and hox genes identified in this study and in an EST database**

**Gene name**	^***a***^**Number of sequences had a hit with nr database**	^***b***^**Number of known sequences from NCBI nucleotide database**	^***c***^**Number of known sequences in an EST sequencing project**[13]
Transforming growth factor-*β*s	6	1	1
Fibroblast growth factors	8	5	2
Hox genes	28	7	1

*a* Number of sequences obtained in this study that have a hit with the corresponding proteins in the NCBI nr database.

*b* Number of sequences from NCBI nr database hit in *a* that show homology with corresponding proteins from NCBI nucleotide database (to July 2012).

*c* Number of sequences that can be identified in an EST database established by an *ambystoma mexicanum* EST sequencing project [13].

**Table 3:** **Identified *transforming growth factor***-***βs***, ***fibroblast growth factors***, **and *Hox *genes**

**Gene name**	**Gene ID**	**Length**	**Subject ID**	**Species**	**E value**
Transforming growth factor-beta-1	Unigene8146_All	3212	gi|160,334,206|gb|ABX24523.1|	Ambystoma mexicanum	0
Transforming growth factor-beta-2	Unigene17537_All	2048	gi|155,966,093|gb|ABU41003.1|	Anser anser	0
Transforming growth factor-beta-3	Unigene9879_All	866	gi|257,173|gb|AAB23575.1|	Gallus gallus	5.00E-19
Fibroblast growth factor-2	Unigene114478_All	512	gi|15,216,301|dbj|BAB63249.1	Cynops pyrrhogaster	9.00E-80
Fibroblast growth factor-8	Unigene116687_All	1666	gi|12,024,873|gb|AAG45674.1|AF190448_1|	Ambystoma mexicanum	1.00E-117
Fibroblast growth factor-9	Unigene82354_All	1419	gi|61,971,458|gb|AAX58114.1|	Didelphis albiventris	1.00E-113
Fibroblast growth factor-10	Unigene32567_All	2235	gi|19,031,193|gb|AAK59700.1|	Ambystoma mexicanum	1.00E-113
Fibroblast growth factor-12	Unigene43917_All	396	gi|238,815,007|gb|ACR 56700.1|	Ovis aries	5.00E-44
Fibroblast growth factor-16	Unigene113330_All	434	gi|224,098,374|ref|XP_002195984.1|	Taeniopygia guttata	7.00E-36
Fibroblast growth factor-17-like	Unigene88737_All	636	gi|301,757,980|ref|XP_002914827.1|	Ailuropoda melanoleuca	4.00E-43
HoxA3	Unigene18117_All	1271	gi|220,898,179|gb|ACL 81435.1|	Latimeria menadoensis	1.00E-115
HoxB3	Unigene92817_All	203	gi|37,528,843|gb|AAQ92347.1|	Pleurodeles waltl	4.00E-16
HoxD3	Unigene38161_All	710	gi|126,341,823|ref|XP_001362908.1|	Monodelphis domestica	7.00E-72
HoxD4	Unigene74557_All	206	gi|301,128,902|emb|CBL59364.1|	Scyliorhinus canicula	3.00E-06
HoxA7	Unigene15254_All	478	gi|6,754,234|ref|NP_034585.1|	Mus musculus	1.00E-27
HoxB8	Unigene107183_All	283	gi|256,014,548|gb|ACU56828.1|	Ichthyophis cf. kohtaoensis JMW-2009	2.00E-18
HoxD8	Unigene1050_All	1896	gi|7,271,820|gb|AAF44632.1|AF224263_2|	Heterodontus francisci	6.00E-95
HoxA9	Unigene115485_All	641	gi|149,633,999|ref|XP_001509421.1|	Ornithorhynchus anatinus	7.00E-72
HoxC9	Unigene108008_All	294	gi|220,898,209|gb|ACL81463.1|	Latimeria menadoensis	4.00E-34
HoxD9	Unigene101797_All	242	gi|118,093,579|ref|XP_001234507.1|	Gallus gallus	3.00E-25
HoxA10	Unigene8140_All	2019	gi|301,607,691|ref|XP_002933440.1|	Xenopus (Silurana) tropicalis	1.00E-127
HoxC10	Unigene115613_All	670	gi|11,037,556|gb|AAG27630.1|AF298185_1|	Ambystoma mexicanum	1.00E-101
HoxD10	Unigene114687_All	531	gi|194,043,944|ref|XP_001925011.1|	Sus scrofa	2.00E-75
HoxC11a	Unigene109718_All	323	gi|301,614,392|ref|XP_002936692.1|	Xenopus (Silurana) tropicalis	3.00E-39
HoxC12	Unigene86529_All	681	gi|24,637,176|gb|AAN63593.1|AF434200_1|	Pleurodeles walti	5.00E-68
HoxA13	Unigene115397_All	626	gi|220,898,176|gb|ACL81432.1|	Latimeria menadoensis	1.00E-103
HoxD13	Unigene116723_All	1926	gi|301,612,439|ref|XP_002935723.1|	Xenopus (Silurana) tropicalis	1.00E-123

### Comparing gene expression profiles between blastema and denervated limb stump

To identify specific genes participating in limb regeneration, the differentially expressed genes between blastema and non-regenerating denervated limb stump were analyzed using 3 different *de novo* assemblers to increase accuracy: Trinity [40], trans-ABySS [41], and SOAP de-novo [42]. BLASTx was utilized to compare the assembled contigs from the 3 respective assemblers with the protein database of *Xenopus laevis*. The contigs with the best e-values were assigned to represent the potential axolotl genes. The fold change of the reads per kilo base of isotig length per million mapped reads (RPKM) values between the libraries from the two tissue types was calculated to identify differentially expressed genes. The numbers of differentially expressed genes for each assembler are: Trinity 2651, trans-ABySS 2693, and SOAP de-novo 2833. Because various assemblers deliver different sets of differentially expressed genes, only genes homologous to a *Xenopus laevis* protein with corresponding contigs from all the 3 assemblers were selected to obtain more accurate results. With this strategy, we detected 1953 genes with differential expression levels between blastema and denervated limb stump libraries. Of these differentially expressed genes, 885 were up-regulated and 1068 were down-regulated in blastema (Additional file 3). Among the up-regulated genes, *anterior gradient* rescues a denervated limb stump to become regenerating again [43]; *matrix metalloproteinase 13* (*mmp13*) is associated with metamorphic developmental processes in axolotl epidermis [44]; *sox9* is essential for sclerotome development and cartilage formation [45]; and *patched1* mediates hedgehog signaling into blastema [46]. The set of differentially expressed genes from this study was compared with previous sequencing projects using 454 pyrosequencing and microarray [14] (Additional file 4). Only the data sets in the previous studies on tissues 14 days after amputation, similar to this study, were chosen for comparison. The unigenes that could not be annotated were excluded here. The Venn diagrams in Additional file 4 reveal overlaps between these 3 data sets. Among the overlapped genes, genes known to be necessary for limb development and limb regeneration were identified including *wnt5a* which is involved in Wnt/planar cell polarity signaling, and *msx2* which plays important roles in the development of limb buds.

### Functional annotation of differentially expressed genes

**Figure 4:**
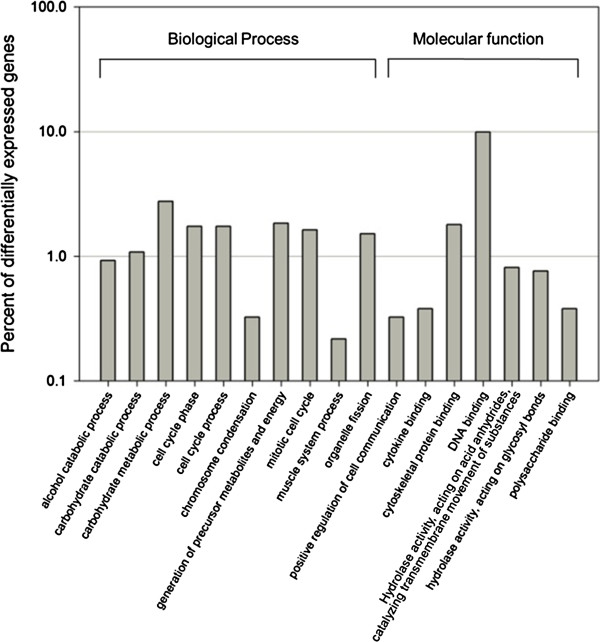
**GO assignment of genes up- or down-regulated in balstema compared with the control non-regenerating limb stump.** The results of GO biological function assignment annotation of differentially expressed genes are summarized in two main categories: biological process and molecular function.

To understand the functions of differentially expressed genes, the set of 1953 differentially expressed genes which had corresponding contigs from all 3 assemblers were mapped to terms in the GO biological process database and compared with the whole transcriptome background to identify genes involved in important biological processes (Figure 4). Among all genes with GO assignment annotations, 1848 differentially expressed genes between blastema and denervated limb stump libraries were annotated. Among the differentially expressed genes, genes involved in nitrogen and DNA metabolism were significantly up-regulated in blastema, such as glucose dehydrogenase, GPI mannosyltransferase, and DNA replication licensing factor mcm. These findings suggest that the cell proliferation rate in axolotl blastema is higher than in denervated limb stumps, a finding consistent with that of a previous report [46].

### Validation of differentially expressed genes by qPCR

**Figure 5:**
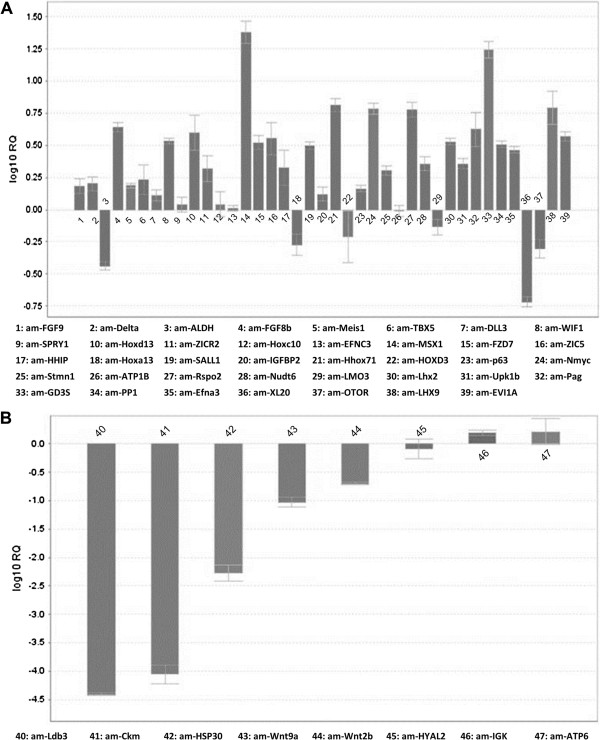
**qPCR validation of the differentially expressed genes related to regeneration.** Thirty nine genes determined as up-regulated **(A)** and 8 genes determined as down-regulated **(B)** in the blastema by the RPKM values were validated using qPCR. Genes are represented by gene names. Bar values represent mean ± SE of three independent measurements.

Among the genes classified as differentially expressed in blastema, 47 regeneration-related genes were selected based on our interests and their differential expression levels were verified by qPCR. Amplification of an endogenous gene, *S21* ribosomal RNA, was used for normalization. Among the 39 genes determined by sequence analysis as up-regulated in blastema, qPCR results confirmed that 33 genes had higher expression levels in blastema (Figure 5A). Results also confirmed a >10-fold higher expression level for *msx1* in blastema. *Msx1* is involved in the regulation of dedifferentiation for mature myofibers. The morpholino-based suppression of Msx1 protein expression in myonuclei significantly inhibits the fragmentation and dedifferentiation of salamander myofibers [47]. In the initially developed limb bud in mice, *msx1* appears in the entire mesenchyme. However, this expression is restricted to the most distal part of the limb bud as development proceeds [48]. When *msx1*-negative proximal limb tissues are grafted into the distal portion of the limb bud, *msx1* is re-expressed in the mesenchyme of the transplanted tissues, which subsequently become dedifferentiated [49]. The homeo box gene *hoxd13* was shown to be expressed at higher levels in blastema. In mice, the *hoxd13* gene plays a key role in axial skeleton development and forelimb morphogenesis [50]. The present sequence analysis shows that *fgf*s were up-regulated in blastema, and this pattern was partly confirmed by showing up-regulation of *fgf*-*8b* and *fgf*-*9* in qPCR analysis. The expression patterns of *fgf*s in this study are consistent with those reported previously [30]. We selected 8 genes classified as down-regulated in blastema to be validated by qPCR. The results confirm that 5 of the 8 genes had lower expression levels in blastema. Among them, *Wnt*-*9a* and *Wnt*-*2b* showed higher expression levels in the denervated limb stump than in blastema (Figure 5B).

### Expression patterns of differentially expressed genes in limb regeneration

**Figure 6:**
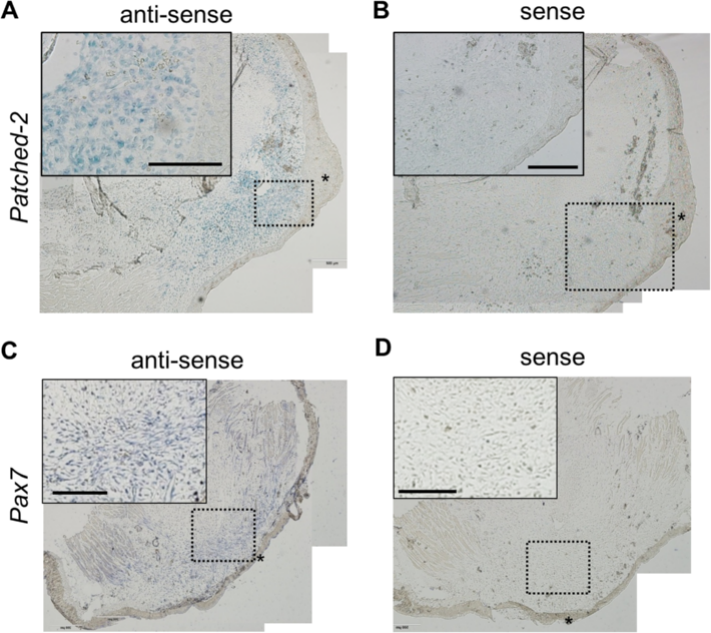
***In situ *hybridization analysis of *patched*-*2 *and *pax7 *expression in the regenerating blastemas.***Patched*-*2***(A)** or *pax7***(C)** signals (blue) using respective anti-sense probes are shown in the distal tip of regenerating limbs. No signal was detected in the negative-control sections using respective sense probes of *patched*-*2***(B)** and *pax7***(D)** on serially sectioned slides. The insets in the left upper are the enlarged photos of respective areas indicated by dotted lines at blastema. Asterisks indicate apical epithelial cap covering the blastema. Scale bars indicate 200 *μ*m.

Because of the technical difficulty of completely isolating blastema from the underlying stump and epidermis, we could not exclude the possibility that the higher expression levels of certain genes in blastema might be attributed to contaminated neighboring tissues. Thus, *in situ* hybridization may reveal the specific cells expressing the genes in this study. The hybridization showed that *patched*-*2*, one of the genes differentially expressed in blastema, was expressed in the cells located in blastema (Figure 6A). Hybridization using the sense control probe showed no signal (Figure 6B). The signal of *patched*-*2* expression was limited to only a portion of blastemal cells, and no signal was shown in the epidermis (Figure 6A). These results confirmed that *patched*-*2* was differentially expressed in blastema, as seen from NGS and qPCR, and that it was expressed in blastemal cells rather than contaminated tissues during tissue sampling. Similarly, *pax7* was also shown expressed in blastemal cells (Figure 6C). Hybridization using the sense control probe showed no signal (Figure 6D). Hedgehog signaling, the signals mediated by activated membrane *patched*-*1* and *2*, is required for blastema growth and control of dorsoventral patterning [51]. During limb development, hedgehog signaling up-regulates *fgf4* to promote limb bud proliferation [52]. Pax7-positive cells also appear in tail blastemas and mid bud-stage limb blastemas [42,53]. Pax7 is required for the specification of myogenic satellite cells, which are myogenic progenitor cells. These results demonstrate that NGS procedures can identify potential markers for the blastemal progenitor cells that initiate limb regeneration.

## Conclusion

This study uses Illumina sequencing technology to sequence the axolotl transcriptomes, perform assembly and annotation on these sequences, and analyze the differentially expressed genes. More than 116,787 distinct sequences were produced with 39,200 sequences below a BLAST cut-off threshold of 10^-5^. Along with various existing axolotl transcriptome databases [13-15], these results can further help researchers investigate axolotl’s limb regeneration. This study also demonstrates the feasibility of using multiple assemblers to increase the identification accuracy of the differentially expressed genes involved in axolotl limb regeneration.

## Methods

### Animal experimental procedures

Wild-type and white mutant (d/d) axolotls were kept at 14–20°C in tap water at pH 6.5-7.5. Juvenile axolotls with an approximate 80 to 90 mm snout to vent length were subjected to denervation at the brachial plexus (third to fifth spinal nerves) of the right upper limbs, leaving the nerve ends a gap of at least 5 mm. After 1 wk, amputation was performed at both forearms at the mid-radius/ulna. At 14 d post-amputation, the full-thickness skin at the tip of limb was gently peeled away by cutting through the full-thickness skin around the circumference of the limb with spring scissors, and the blastemas in the left forearms and non-regenerating stump tissues in the right forearms were collected for total RNA extraction. All the surgical experiments were conducted under anesthesia with 0.1% MS-222 (Sigma-Aldrich, St. Louis, MO, USA). Animal care and experimental procedures were approved by the Institutional Animal Care and Use Committee of National Taiwan University College of Medicine.

### RNA extraction and library preparation

Total RNA was extracted using Trizol Reagent (Invitrogen, Carlsbad, CA, USA) and isolated by RNeasy mini-columns (Qiagen, Germantown, MD, USA). RNA quality was assessed by spectrophotometry. A fragmentation buffer (100 mM ZnCl_2_ in 100 mM Tris-HCl pH7) was added to cut mRNAs into short fragments. Fragmented RNA was reverse-transcribed to first-strand cDNA with reverse transcriptase (Invitrogen) in the presence of a random hexamer-primer (Invitrogen) and dNTPs for 50 min at 42°C. The second-strand cDNA was synthesized using DNA polymerase I in a buffer containing dNTPs and RNaseH. The short DNA fragments were purified using the QiaQuick PCR extraction kit (Qiagen) and resolved with an elution buffer (10 mM Tris-Cl, pH 8.5) for end reparation and addition of a poly(A) fragment to both ends. Thereafter, the short fragments were connected with sequencing adapters and then separated in gels by electrophoresis. The fragments with a size suitable for NGS were cut from gels and eluted for PCR amplification by using adapter primers.

### Analysis of Illumina sequencing results

Four analytic procedures were conducted on the RNA-seq data.

#### Illumina sequencing and de novo assembly

The 2 cDNA libraries were sequenced from both the 5' and 3' ends on the Illumina GA II following the manufacturer’s instructions. The low-quality raw sequences were removed. The remaining short reads with their adaptor sequences trimmed were assembled in a *de novo process* using 3 assemblers: Trinity [40], trans-ABySS [41], and SOAP de-novo [42]. Similar assembly parameters were used for the 3 assemblers to compare performance. Trinity and SOAP de-novo used the default k-mer setting [40,42], and trans-ABySS was run using k-mer values of 45, 46, to 89 [41]. All assemblies were performed on a server with 24 cores and 128 GB of memory. After assembly, the contigs longer than 100 bases were used for subsequent analysis.

#### Functional annotation

BLASTx [53] was performed to align the assembled contigs from the 3 assemblers to the nr protein database for functional annotation. The e-value cut-off was set at 1E-5 for further analysis. Each assembled contig was assigned with the gene name and related function based on the best BLASTx hit (the smallest e-value). Assembled contigs assigned to the same gene were further compared, and the contig from the best e-value was adopted. If there was a tie between 2 assembled sequences, the one with the largest sequence identity was selected. In the end, with respect to each assembler, a unique contig was used to represent a potential gene of axolotl, and this gene was named by the corresponding protein in *Xenopus laevis*.

#### Quantization of transcript sequences

Quantitative analysis was performed using CLCbio (CLC Genomics Workbench 4.8, parameter settings: minimum length fraction, 0.5; minimum similarity fraction, 0.95; maximum number of hits for a read, 10). The reads from 2 libraries were mapped to the selected assembled contigs for various assemblers. The read counts accumulated on the selected contigs were further normalized as RPKM values.

#### Identification of differentially expressed genes

The fold change of RPKM values (the RPKM is replaced with 0.01 if it equals zero) from the libraries of the two tissue types was calculated to identify differentially expressed genes. Because diverse assemblers deliver different sets of potential axolotl genes, only those homologous to a *Xenopus laevis* protein and having corresponding contigs from all the 3 assemblers were further analyzed. The genes with 2-fold up- or down-regulation that were consistent among the 3 assemblers were identified.

### qPCR for mRNA quantification

RNA was prepared using Trizol Reagent (Invitrogen). The RNA samples of the blastema tissue and denervated stump end were harvested from the upper limbs 14 days after amputation. The total RNA was reverse-transcribed to first-strand cDNA with reverse transcriptase (Invitrogen) in the presence of a random hexamer-primer (Invitrogen) and dNTPs for 50 min at 42°C. The expression levels of specific mRNAs were determined by qPCR using respective primer pairs (Additional file 5). Each reaction was conducted in a total volume of 20 *μ*L containing 50 ng first-strand cDNA, 10 *μ*L 2X Fast SYBR^®^ Green Master Mix (Applied Biosystems, Carlsbad, CA, USA), and each primer pair at 0.5 *μ*M. A Step One™ Real-Time PCR system (Applied Biosystems) was used. Data was analyzed using Step One™ software version 2.1 (Applied Biosystems). Error bars indicate RQ max and RQ min.

### *In situ* hybridization

Templates of cDNA for axolotl *patched*-*2*(319 bp) and *pax7* (303 bp) were amplified by RT-PCR using respective sense primers linked to SP6 promoter sequence and anti-sense primers linked to T7 promoter sequence. Sense and anti-sense RNA probes were synthesized from the cDNA templates using a digoxigenin RNA labeling kit following the manufacturer’s protocol (Roche, Indianapolis, IN, USA). RNA probes were prepared using respective primers: *patched*-*2* (anti-sense), 5' CGATTTAGGTGACACTATAGAAGAGTCCAACGACGTGAGGACAAGA- 3'; *patched*-*2* (sense), 5'- CGTAATACGACTCACTATAGGGAGATTGAGCTGGATGGCGTGAA-3'; *pax7* (anti-sense), 5'-CGATTTAGGTGACACTATAGAAGAGAAACAGGCAGGAGCCAATCAA-3'; and *pax7* (sense), 5'-CGTAATACGACTCACTATAGGGAGAGCTGACCGGATTCATGTGGTT-3'.

Blastema tissues were fixed overnight at 4°C in 4% paraformaldehyde in 1× phosphate buffered saline (PBS) and subsequently embedded in Tissue-Tek (Thermo Scientific, Miami, FL, USA) and frozen at −80°C until sectioning at 10 *μ*m. Before hybridization, sections were digested with 1 *μ*g/mL proteinase K in 1× PBS at room temperature for 8 min, fixed again in 4% paraformaldehyde in 1x PBS at room temperature for 20 min, and carbonated with diethypyrocarbonate in 1× PBS. The slides were covered with Hybri-well Press-seal hybridization chambers (Sigma-Aldrich) and hybridized overnight at 58°C with pre-denatured DIG-labeled probes in a hybridization solution (Roche). After hybridization, the slides were washed in 5× SSC twice, each for 1 h, at 65°C, and then in 0.1× SSC for 30 min at room temperature. The slides were rinsed in a buffer containing 100 mM Tris-HCl (pH 7.5) and 150 mM NaCl. The slides were incubated at 4°C overnight in the same buffer containing an alkaline phosphatase-conjugated anti-digoxigenin antibody (Roche) diluted at 1:1000. The slides were washed in PBST (0.1% TritonX-100 in PBS) 3 times for 30 min each at room temperature. The signals of alkaline phosphatase activities bound on the anti-DIG-antibody were detected using a mixture of the BCIP/NBT solution (Sigma-Aldrich).

## Abbreviations

ESTs: Expression sequence tags; NCBI: National Center for Biotechnology Information; NGS: Next generation sequencing; qPCR: Quantitative polymerase chain reaction; GO: Gene ontology; COG: Clusters of orthologous groups; BLAST: Basic local alignment search tool.

## Competing interests

We declare that we do not have competing interests.

## Authors’ contributions

CHW conducted the molecular genetic studies, participated in the sequence alignment, and drafted the manuscript. CCH participated in the sequence alignment. CYC participated in the design of the study and performed the statistical analysis. MHT conceived the study, participated in its design and coordination, and helped draft the manuscript. HSL designed and coordinated the studies and wrote the manuscript. All authors read and approved the final manuscript.

## Supplementary Material

Additional file 1 **Overview of *Ambystoma mexicanum *transcriptome sequencing and assembly.** Length distribution of blastema contigs (A), DN contigs (B), blastema scaffolds (C), DN transcribed sequences (D), blastema unigenes (E), DN unigenes (F), and all unigenes (G). DN = denervated limb stump. Click here for file

Additional file 2 **Unigenes with a cut-off E-value of 10**^**-5**^**using BLASTx against the non-redundant (nr) NCBI nucleotide database.** Click here for file

Additional file 3 The 885 unigenes determined up-regulated and 1068 unigenes determined down-regulated in blastema. Click here for file

Additional file 4 **Comparisons of the identified differentially expressed genes between this study and previous sequencing and microarray data.** The “Up” and “Down“ spreadsheets include all 885 up-regulated and 1068 down-regulated genes, respectively. The “Up signature” and “Down signature” spreadsheets include only those genes able to be annotated, which are based for comparison in the Venn diagrams. Click here for file

Additional file 5 Primer pairs used in qPCR for validating the differentially expressed genes. Click here for file
