# Supplementary figures and images for: De novo transcriptome sequencing of axolotl blastema for identification of differentially expressed genes during limb regeneration
Source: BMC Genomics. 2013 Jul 1;14:434. doi: 10.1186/1471-2164-14-434 (PMC3702472; doi:10.1186/1471-2164-14-434)

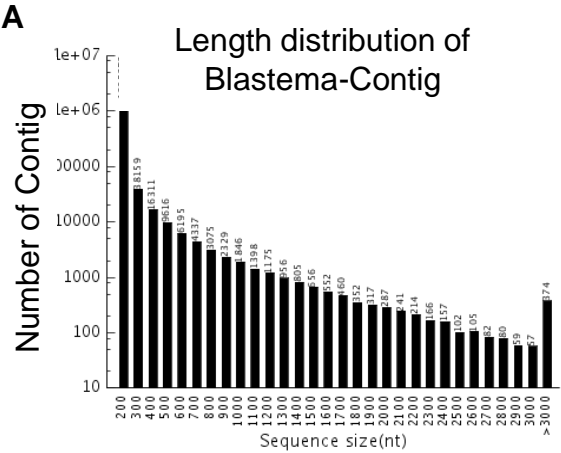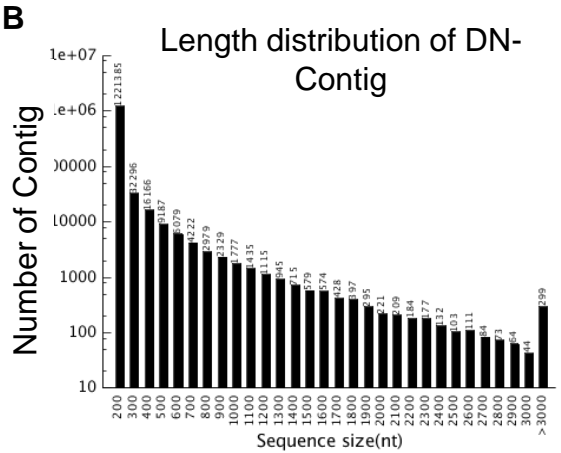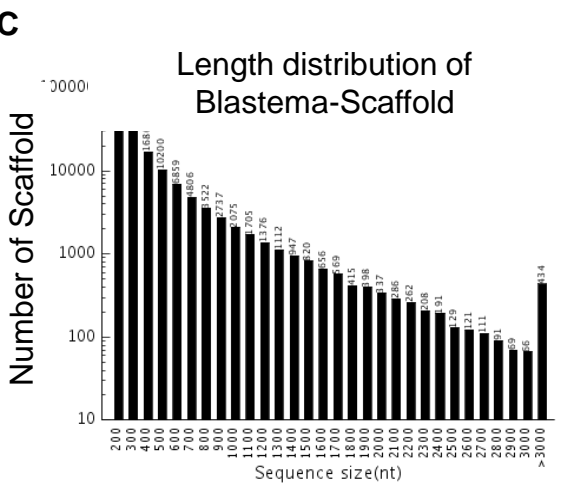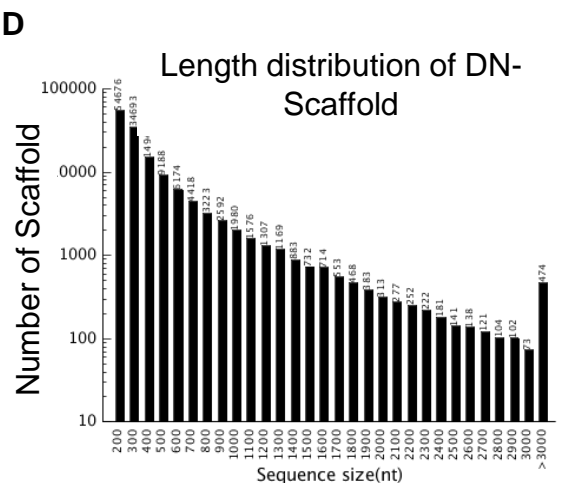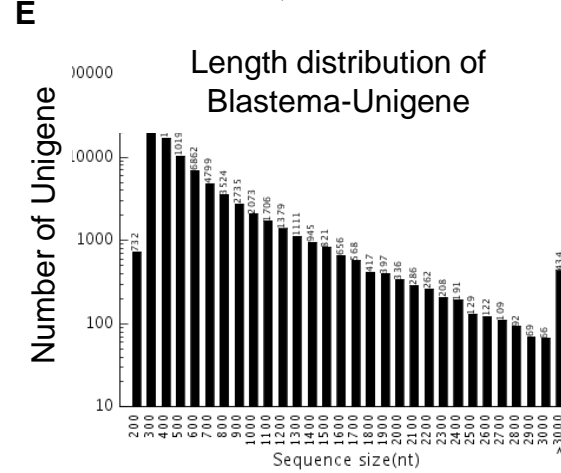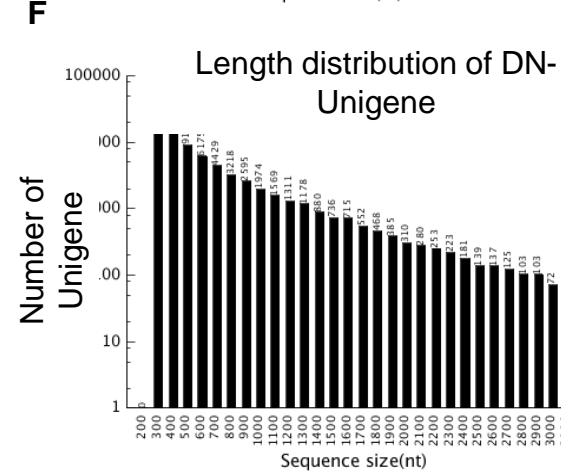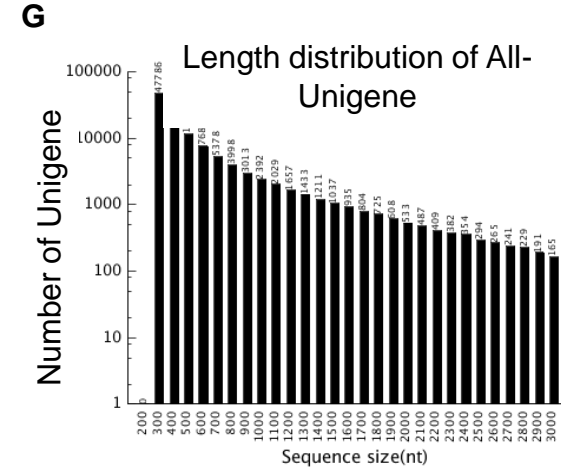

Supplement: Additional file 1 — Overview of Ambystoma mexicanum transcriptome sequencing and assembly. Length distribution of blastema contigs (A), DN contigs (B), blastema scaffolds (C), DN transcribed sequences (D), blastema unigenes (E), DN unigenes (F), and all unigenes (G). DN = denervated limb stump. [file 1471-2164-14-434-S1.pdf]
